# Mechanical Fatigue of Titanium Dental Implants After Implantoplasty: An In Vitro Study Combined with Finite Element Simulations

**DOI:** 10.3390/jfb17050221

**Published:** 2026-05-02

**Authors:** Esteban Padullés-Roig, Pablo Sevilla, Eugenio Velasco-Ortega, Miguel Cerrolaza, Darcio Fonseca, Jeanne Parache, Conrado Aparicio, Javier Gil

**Affiliations:** 1 Faculty of Dentistry, Universidad Internacional de Catalunya, Josep Trueta s/n, Sant Cugat del Vallés, 08195 Barcelona, Spain; epadu56@gmail.com; 2 Bioinspired Oral Biomaterials and Interfaces, Department Ciencia e Ingenieria de Materiales, Escola Enginyeria Barcelona Est, Universitat Politècnica de Catalunya, Av. Eduard Maristany 16, 08019 Barcelona, Spain; psevilla@euss.cat (P.S.); darciofonseca@beclinique.pt (D.F.); parache.jeanne@estudiantat.upc.edu (J.P.); conrado.aparicio@upc.edu (C.A.); 3 Mechanics Department, Escuela Universitaria Salesiana de Sarria, Pg. Sant Joan Bosco, 74, 74, 08017 Barcelona, Spain; 4 Department of Stomatology, Faculty of Dentistry, University of Seville, c/Avicena s/n, 41009 Sevilla, Spain; evelasco@us.es; 5 School of Engineering, Science & Technology, Valencian International University, Calle Pintor Sorolla, 21, 46002 Valencia, Spain; miguel.cerrolaza@upc.edu; 6 ICREA, Institució Catalana de Recerca i Estudis Avançats, Generalitat de Catalunya, 08010 Barcelona, Spain

**Keywords:** implantoplasty, dental implants, peri-implantitis, fatigue, fracture, finite element simulation

## Abstract

The increasing prevalence of peri-implantitis has led to a growing clinical use of implantoplasty, a procedure involving intraoral machining of the dental implant surface to remove biofilm. The absence of standardized clinical protocols may contribute to premature fatigue failure of dental implants. The present study aimed to evaluate the influence of machining depth on the cyclic mechanical behavior of dental implants. A total of 250 commercially pure grade 4 titanium dental implants were distributed into four groups according to machining depth: untreated (original), 0.2 mm, 0.4 mm, and 0.6 mm wall reduction. The implant system featured an internal connection with a thread height of 0.4 mm. Finite element analysis was performed for each machining depth to evaluate von Mises stress distribution and simulate fatigue behavior. The numerical models were validated through experimental fatigue testing using a servo-hydraulic MTS Bionix testing machine under ISO 14801:2016 conditions, showing a high correlation between simulated and experimental results (correlation coefficients > 0.9). The results indicated that maximum von Mises stresses were concentrated at the junction between the implant thread and the implant body. The fatigue limit of the untreated implants was approximately 351 N. Implants subjected to 0.4 mm machining exhibited a fatigue limit of 301 N, whereas lower fatigue limits were observed for 0.2 mm (255 N) and 0.6 mm (185 N) reductions. These findings suggest a significant mechanical effect of thread removal: 0.4 mm implantoplasty may provide improved fatigue performance compared to 0.2 mm, potentially due to reduced stress concentration at the thread–body junction. At high applied loads, fracture occurred in the coronal region of the implant, whereas at lower loads failure shifted to the implant–abutment connection. Although a good agreement between numerical and experimental results was observed, these findings should be interpreted with caution due to the in vitro testing conditions and the assumptions inherent to the finite element simulations. Therefore, while the results suggest that implantoplasty depth should not exceed the original thread height, further validation under clinically relevant conditions is required to confirm its impact on long-term mechanical reliability.

## 1. Introduction

Peri-implantitis is caused by an inflammatory process triggered by bacterial colonization around the dental implant with the formation of biofilms [1,2]. The consequent persistent inflammation results in bone loss, which ultimately causes the loss of both mechanical and biological fixation of the dental implant [3,4,5]. Several scientific dental societies have reported that approximately 24% of dental implants develop peri-implantitis within the first 10 years of function [6,7]. In order to reduce these high prevalence rates, preventive strategies have been emphasized, particularly oral hygiene measures and recommendations to avoid smoking and alcohol consumption [2,8]. However, peri-implant diseases are also influenced by the patient’s genetic susceptibility [9].

**Figure 1:**
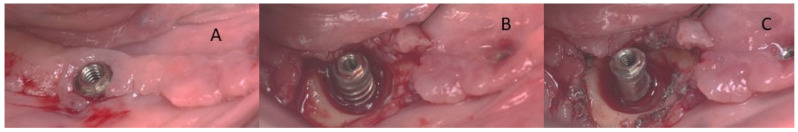
(**A**) Dental implant presenting inflammation in the soft tissues due to peri-implantitis. (**B**) Bone loss caused by the presence of biofilms. (**C**) Dental implant that has undergone mechanization for the removal of biofilms. The loss of roughness and threads of the dental implant can be seen. The presence of titanium particle debris resulting from mechanization can also be observed.

The main problem associated with peri-implantitis is bone loss and the difficulty of eliminating biofilms adhering to the implant surface. In principle, the standard approach when removal of the infected dental implant is necessary would involve disinfection of the surrounding tissues, placement of a calcium phosphate-based biomaterial to induce bone regeneration, and subsequent placement of a new dental implant once regeneration has occurred [10,11]. In many cases, surgery becomes more complex, as placement of a new implant may require extraction of adjacent teeth due to anatomical limitations [12]. This surgical complexity has led to the development of an alternative surgical approach that proposes an aggressive mechanical biofilm removal technique known as implantoplasty. This technique involves mechanical modification of the implant surface intraorally using clinical rotary instruments, particularly highly abrasive burs such as diamond or tungsten carbide burs [13,14,15]. Subsequently, the machined surface is thoroughly cleaned using disinfecting agents such as hydrogen peroxide, sodium hypochlorite, among others [16] (Figure 1).

Implantoplasty is associated with several adverse effects, including loss of surface roughness that promotes osseointegration, reduced corrosion resistance, deterioration of static and cyclic mechanical properties, and the release of particles ranging from nanometric to millimetric sizes. These particles have been shown to exert in vitro cytotoxic effects, particularly by particles from dental implants manufactured with the Ti6Al4V alloy [17]. Particle size is a critical factor, as nanoparticles are not recognized by the immune system and their effects on the human body remain unknown [18]. Larger particles, however, may be phagocytosed by macrophages or encapsulated by soft tissue [18]. Kotsakis et al. [19] simulated anaerobic inflammatory conditions in the oral cavity and observed oxygen depletion in the environment, leading to the death of aerobic cells, but not anaerobic cells, which are the most pathogenic. Oxygen depletion causes reduction in the titanium oxide layer of the implant to pure titanium, thereby compromising the passive layer that protects the implant surface. When inflammation subsides, oxygen levels in the area recover, reinducing oxidation of pure titanium; however, this oxidation does not result in stoichiometric titanium dioxide but rather in non-stoichiometric mixed oxides that are not cytocompatible. Another major concern is the loss of mechanical properties of dental implants due to machining, as a significant reduction in implant cross-section occurs [14]. This aspect has been poorly studied, particularly with regard to mechanical behavior under cyclic loading.

As described above, implantoplasty is a procedure associated with numerous drawbacks; however, it is currently widely used due to its effectiveness in infection control and its ability to avoid lengthy procedures for the patient, as well as the high cost associated with implant removal, tissue disinfection, regeneration, and placement of a new dental implant. Various methods have been proposed to avoid titanium machining during implantoplasty, such as electrolysis using the dental implant itself as an electrode, generating hydrogen gas bubbles that remove the biofilm [20]. Fonseca et al. [21] demonstrated the effectiveness of this approach in biofilm removal without altering the titanium properties of the dental implant. Other methods, still under experimental development, include pressurized water jet treatments [21]. Antibiotic therapies and aggressive chemical agents have been discarded, as pharmaceuticals are ineffective in eliminating biofilms and promote antibiotic resistance, while aggressive chemical agents may induce tissue necrosis and acid attack on titanium, increasing surface roughness and thereby favoring subsequent bacterial recolonization of the implantoplasty-treated surface or limiting re-osseointegration of the affected implant [22]. Similarly, ozone treatments do not eliminate biofilms and may cause necrosis of oral soft tissues when applied for prolonged periods [23,24].

Although implantoplasty has been proposed as an effective adjunctive approach for the management of peri-implantitis, its clinical application remains a matter of debate. While several studies suggest that surface smoothing may reduce bacterial adhesion and facilitate maintenance, concerns persist regarding potential adverse effects, including alterations in implant surface integrity, reduction in mechanical strength, and the possible release of metallic particles. Furthermore, alternative therapeutic strategies—such as resective surgery without implant modification, regenerative approaches, or the use of adjunctive chemical and mechanical decontamination methods—continue to be widely investigated, with no clear consensus on a gold-standard treatment.

From a biomechanical perspective, the evidence remains limited and, in some cases, inconsistent. In particular, the impact of implantoplasty on fatigue behavior and long-term mechanical stability has not been sufficiently clarified, despite its direct clinical relevance. Most available studies focus on surface characteristics or short-term outcomes, leaving a significant gap regarding the structural consequences of material removal under functional loading conditions. Therefore, a more comprehensive evaluation of the biomechanical implications of implantoplasty, especially under fatigue conditions, is needed to better inform clinical decision-making.

There is currently a debate among clinicians regarding the appropriateness of implantoplasty in elderly patients, in cases that do not compromise adjacent teeth, and among other clinicians, who view implantoplasty as a surgical procedure that causes a loss of mechanical properties, increased corrosion rates, and the release of particles ranging in size from nanometers to millimeters, along with the potential effects these particles may have on adjacent tissues.

On the one hand, implantoplasty removes bacterial biofilms, halts bone loss, and slows peri-implantitis. Clinical evidence from patients is favorable, and in many cases, the procedure resolves the issue satisfactorily. However, there is scientific evidence indicating that implantoplasty leads to a loss of titanium properties and causes inflammation around the released particles, with cases of metallosis observed in some instances. The lack of implantoplasty protocols and scientific studies evaluating the consequences of implantoplasty causes confusion among clinicians. In this context, the objective of this study is to address this gap by conducting research and contributing new insights into the fatigue behavior of implants subjected to implantoplasty. The specific objective of this study is to investigate the influence of the reduction in the implant’s cross-sectional area due to machining on its cyclic mechanical behavior by reducing it by 0.2, 0.4 (height of the implant thread), and 0.6 mm. A finite element simulation of the mechanical behavior was performed, and the results were validated with mechanical fatigue tests. These results can help dentists determine a safe level of cross-sectional reduction that prevents fatigue fracture of dental implants during use. The null hypothesis is that cross-sectional reduction does not influence fatigue behavior.

## 2. Materials and Methods

**Figure 2:**
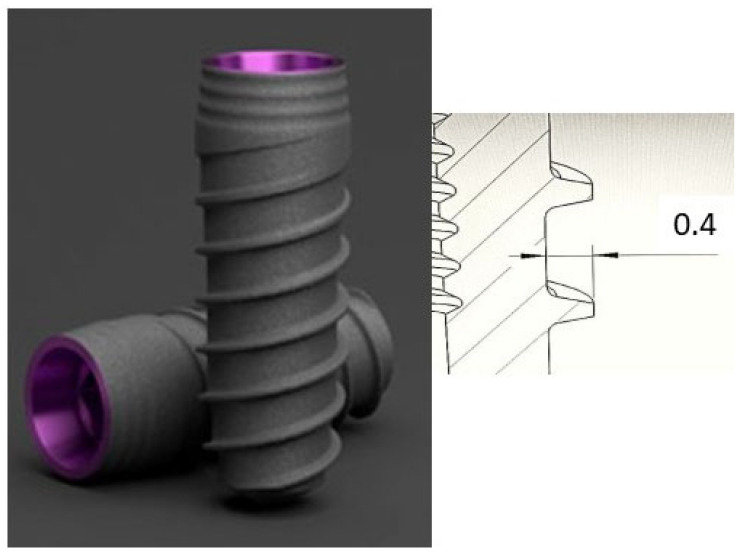
Dental implants studied and scheme of the design of the thread in mm.

A total of 250 dental implants of 4 mm in diameter (Vega, Klockner, Escaldes Engordany, Andorra) were studied. The dental implants were manufactured from commercially pure grade 4 titanium. The roughness (Sa) of the surface was 0.9 ± 0.2 μm. The height of the thread was 0.4 mm. In Figure 2 the dental implants studied and the scheme of the thread can be observed.

**Figure 3:**
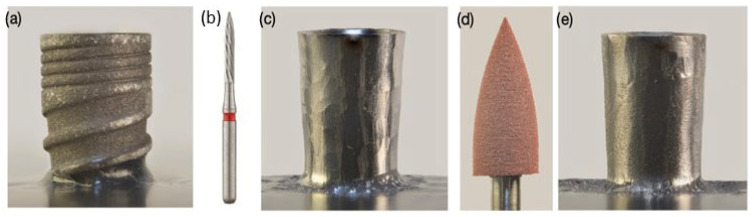
(**a**) Original dental implant. (**b**) Drill of tungsten carbide. (**c**) Implant machined by the drill of tungsten carbide. (**d**) Drill of particles of rubber to polish the surface. (**e**) Dental implant machined after the implantoplasty.

Control samples in the as-received condition were characterized, along with four additional sample sets corresponding to distinct implantoplasty procedures. The implantoplasty procedure was carried out by a single dental clinician (E.P.G) to ensure consistency in the treatment protocol and to reduce variability in both surface finish and experimental outcomes. The procedure was carried out using a force-controlled dynamometer to apply consistent pressure between the drill and the titanium surface. The applied force was maintained at a constant load of 10 N using an automatic dynamometric control system, corresponding to the mean force measured by 59 clinicians at the Universitat Internacional de Catalunya’s University Dental Clinic. The application of the load lasted 60 s for each disk. The surface of the samples was sequentially modified using a GENTLEsilence LUX 8000B turbine (KaVo Dental GmbH, Biberak, Germany) with continuous irrigation, the surface was sequentially modified with a fine-grained tungsten carbide bur (reference H481. 312.014 KOMET; GmbH & Co. KG, Lemgo, Germany) and a fine-grained rubber polisher (order No. 9618.314.030 KOMET; GmbH & Co. KG, Lemgo, Germany). (Figure 3). In all cases, the dental implants undergoing implantoplasty were continuously irrigated with demineralized water at 25 °C at a flow rate of 8 mL/s using the Dentsply-Sirona Axano 323 system (Auckland, CA, USA).

Four groups were studied: control (as-received implant) and three groups machined as implantoplasty at three depths: 0.2, 0.4 (height of the thread) and 0.60 mm. To ensure the thicknesses, all implantoplasties performed were examined under a stereoscopic magnifying glass (Zeiss 400Sb-V. Berlin, Germany) with a resolution of 0.01 mm. Implantoplasties were performed and were selected for each section reduction to ensure the section. The section was measured by stereoscopic magnifier (Olympus, T2400, Tokyo, Japan) in all surfaces and were tested when the differences in the section were ±0.01 mm. Of the 250 dental implants, 200 were selected for mechanical property testing. The selection criteria were based on the extent of the implantoplasty performed. The groups were:

50 original dental implants as controls.

50 dental implants with 0.2 mm of implantoplasty.

50 dental implants with 0.4 mm of implantoplasty.

50 dental implants with 0.6 mm of implantoplasty.

Of the 50 dental implants, 10 were used to determine the flexural strength and the remaining 40 dental implants were subjected to fatigue testing. To determine the sample size, the following method was used: ‘Inference for Means: Comparing Two Independent Samples’ website developed by the Department of Statistics at the Univ. of British Columbia (http://www.stat.ubc.ca/~rollin/stats/ssize/n2.html) (accessed on 25 February 2025) [25]. A sample size of 7 was calculated for a desired power of 0.80 and significance of *p*-value < 0.05. In this work, we indeed tested 10 samples, which is a sample size larger than the minimum necessary for the desired power. This is because the maximum flexural strength value determines the load percentages at which fatigue tests must be conducted. Given the importance of this value, we increased the number of samples to 10.

### 2.1. Finite Element Analysis

A three-dimensional model of the 4 mm Klockner Vega implant was provided by the manufacturer. Modifications were implemented on the model utilizing Altair Inspire 2024 software to accurately simulate the abutment employed in in vitro assays and the implantoplasty procedures.

Model meshing and subsequent static analyses were conducted using HyperMesh software, employing Altair’s Optistruct solver (version 3.1). Fatigue analyses were performed utilizing Hyperlife software (version 2.0), also developed by Altair. To ensure the accuracy and reliability of the numerical results, a mesh convergence study was performed. Mesh independence was achieved when the peak von Mises stress varied by less than 2% between refinements. The final domain was discretized using 4-node linear tetrahedral elements (CTETRA4 in HyperMesh). The mesh utilized a global element size of 0.25 mm, with a localized refinement down to 0.05 mm in critical areas, specifically at the implant threads and the regions subjected to maximum bending stress. To evaluate the overall bending behavior and the critical stresses at the implant walls resulting from the implantoplasty, the implant and the abutment were modeled as a single continuous solid body, simplifying the connection as a perfectly rigid interface. Boundary conditions were applied by fully constraining the surface nodes below the embedding plane. There were no contact conditions between implant components in this FEA model because the implant and abutment were modeled as a single, monolithic solid. This continuous solid approach was chosen deliberately to isolate the macroscopic bending stresses and the structural weakening caused by the implantoplasty, without introducing the complex frictional micro-mechanics of the screw joint.

**Figure 4:**
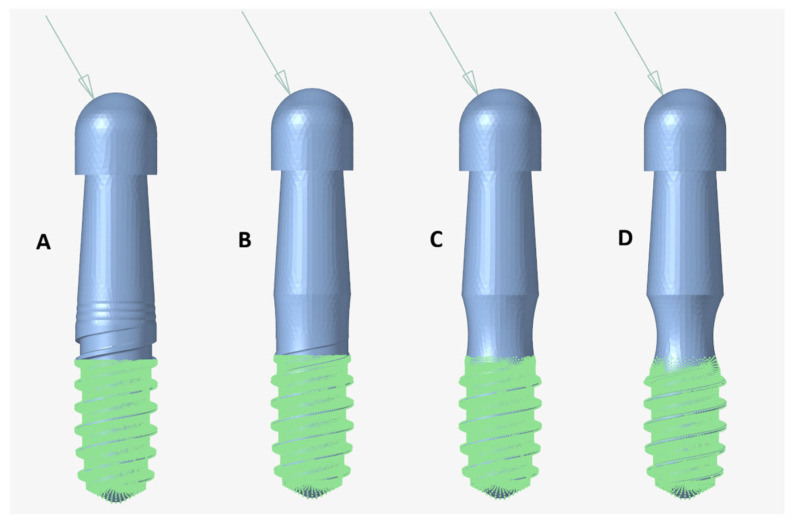
Modelization of dental implants with different depth of machining. (**A**) Original implant. (**B**) Dental implant with implantoplasty of 0.2 mm. (**C**) Dental implant with implantoplasty of 0.4 mm. (**D**) Dental implant with implantoplasty of 0.6 mm. The green color indicates the part in contact with the bone, and the blue color indicates the part that is not osseointegrated.

The static simulation analysis was performed following the conditions stipulated by the UNE EN-ISO 14801 standard [26]. This standard specifies the application of an external load at a 30° angle, 8 mm from the nominal bone level, with the implant embedding plane situated 3 mm below this reference level. Figure 4 illustrates the shape of the different implants and the geometric configuration applied during the static analysis.

The applied load in the static analysis was set to 550 N, corresponding to the load used for the static analysis to compute stress distribution for the S-N curve calculation. The implant material was modeled as an isotropic bilinear elastoplastic material, characterized by Young’s modulus of 106 GPa, a Poisson’s ratio of 0.37, and a yield strength of 550 MPa, based on the manufacturer’s specifications. Post-yield behavior in the plastic region was modelled linearly, with the slope defined by the tangent modulus [27]. The tangent modulus to simulate hardening after the yield point was determined using the Ramberg–Osgood equation [28], by employing a strain hardening exponent of 0.1 and a strength coefficient of 1.000 MPa [29]. This provided a tangent modulus of 2.765 MPa, which was subsequently utilized in the finite element analysis (FEA).

Fatigue life prediction was conducted using Altair HyperLife. Given that the dental implants are designed to withstand millions of masticatory cycles, the analysis was focused on the high-cycle fatigue (HCF) regime. Therefore, a nominal stress-life (S-N) approach was adopted. The baseline S-N curve for the commercially pure grade 4 titanium was synthesized using the material’s ultimate tensile strength (UTS = 680 MPa). Since the experimental cyclic loading was applied with a stress ratio of (pulsating load), the Goodman mean stress correction model was implemented [6]. This correction is critical to properly account for the detrimental effect of the non-zero tensile mean stress on the fatigue life of the material. The signed von Mises equivalent stress tensor, extracted from the elastoplastic static load step, was utilized as the primary damage parameter to compute the fatigue cycles to failure.

The Goodman’s criterion was adopted herein as it provides a reliable and conservative estimation for ductile materials, such as the titanium Grade 4 used in this simulation. Unlike the Gerber criterion [30], which may underestimate the effect of mean tensile stresses, or the Soderberg approach [31], which is often excessively restrictive by relying on the yield strength, the Goodman relation effectively balances clinical safety with mechanical reality. This is particularly relevant in dental implantology, where implants are subjected to complex cyclic loading under pre-tension due to screw tightening. By incorporating the Ramberg–Osgood parameters [28] into the plastic behavior model, the FEA results account for the strain-hardening effects that occur after the yield point, ensuring that the stress distribution used for the S-N analysis reflects the true structural response of the material under maximum functional loads.

The finite element simulation performed includes certain assumptions in its application that limit the interpretation of the results obtained and must be taken into account. One of these assumptions concerns the connection between the titanium and the bone, which is assumed to be complete; the simulation treats this as if osseointegration were total, whereas it is known that bone–implant contact is generally between 65% and 70%. This fact causes the simulation to overestimate the transfer of mechanical loads compared to reality. Another assumption is that the finite element analysis does not account for the residual compressive stress of the dental implant, which would lead to an increase in stress on the implant when it is mechanically loaded, nor does it account for possible microcracks or small drill debris that may remain on the surface of the dental implant. These defects can affect load transmission or stress concentration on the surface of the dental implant, which may impact the simulation.

The results of the finite element simulation were validated by calculating the regression coefficients of the straight lines fitted to the data points from the fatigue tests. To perform these adjustments and determine the correlation coefficients with the experimental data, the MATLAB-Simulink software was used, specifically the polyfit and polyval packages, version 2.0 and 2.2, respectively (Natik, MA, USA).

### 2.2. Fatigue Testing

**Figure 5:**
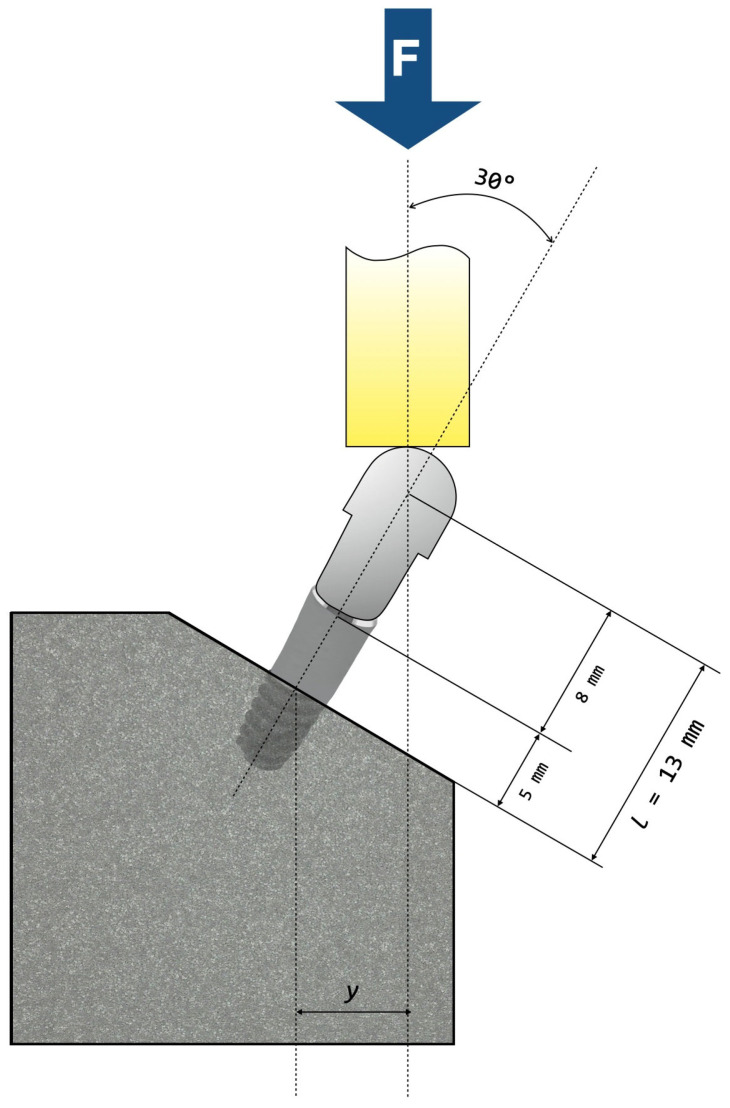
Schematic representation of the test setup according to ISO 14801:2016, except for nominal bone level.

We carried out fatigue testing in room air and at room temperature using a servo-hydraulic mechanical testing machine (MTS Bionix 370, MTS^®^, Eden Prairie, MN, USA) equipped with a 15 kN load cell (MTS Load Cell 661.19H-03, MTS^®^, Eden Prairie, MN, USA). We screwed identical hemispherical abutments to each implant with the torque recommended by the manufacturer (35 N·cm). The loading center was located 13 mm above the resin (nominal bone level). According to ISO 14801:2016 [26], we placed the samples in a stainless-steel clamping jaw so that loading had an angle of 30° to the longitudinal axis of the implant (Figure 5).

According to European Standard [26], the general principles for fatigue testing state that “at least two, and preferably three, specimens shall be tested at each of at least four loads”. Moreover, “at least three specimens shall be tested, and every specimen shall reach the specified number of cycles with no failures” in order to reach the infinite life range.

To conclude, it should be noted that while this international standard simulates the functional loading of an endosseous dental implant under “worst case” conditions, it is not applicable for predicting the in vivo performance of an endosseous dental implant or dental prosthesis, particularly if multiple endosseous dental implants are used for a dental prosthesis.

Each specimen received a maximum of 5,000,000 cycles of a uniaxial load, perpendicular to the tangent of the dome of the hemispherical abutment. The loading range was between a maximal nominal value and 10% of this value (R = 0.1). To minimize the vibrations of the testing machine, the sinusoidal load frequency was kept at 15 Hz. We used TestStar II^®^ software (MTS^®^, Eden Prairie, MN, USA) (version 3.1) to record data in real time.

The maximum flexural strength of each of the different implant groups is determined by applying a load until failure using the same experimental fatigue testing system specified in the standard. This value corresponds to the zero-cycle fatigue value. In accordance with ISO 14801:2016 [26], tests were carried out by applying a minimum of four series of loads, the first of which was equivalent to 80% of the maximum flexural force (F_maxC_ and F_maxIP_). According to the ISO standard, the minimum number of cycles for infinite lifespan is 5 million cycles if the asymptote is reached on the curve. In our case, tests were conducted up to 10 million cycles to ensure that the asymptote was reached and to guarantee to clinicians that, under these conditions, the dental implants can be considered to have an infinite life.

If any of the samples collapsed before reaching the specified number of cycles, the procedure was started again with two new implants and under a lower load (20% if ≥60% F_max_ and 10% if <60% F_max_). When two consecutive samples reached 5 × 10^6^ cycles without failure, an additional test was performed with a third sample. If the latter did not fail (i.e., 3 consecutive samples without apparent failure), this point was considered to be the fatigue limit beyond which the implant could withstand an infinite number of loading cycles. In case the fatigue limit was reached in fewer than four load series, additional levels (1, 2 or 3) were established by applying a load 5% higher than the previous one. The number of cycles and the state (i.e., intact or failed) of each tested specimen were recorded. Failure was defined as the elastic limit of the material, permanent deformation, loosening of the implant assembly, or fracture of any component. The results of the fatigue tests were displayed in a load versus number of cycles plot (i.e., S-N curve or Wöhler’s curve), which represents the number of load cycles of each sample (logarithmic scale) and the corresponding maximal load (linear scale).

Fractography was studied by means of scanning electron microscopy (JEOL 6400, JEOL, Tokyo, Japan) with X-ray microanalysis (Oxford F300, Oxford Instruments, Abingdon, UK).

The sample size was determined using an experimental approach. To determine the sample size, a design of experiments was used to calculate the number of dental implants to be tested for flexural strength, which turned out to be five. Because fatigue is a statistical issue and, in this case, there are different variables—such as wall reduction due to implantoplasty, applied mechanical loads, as well as other variables that are not exact, such as the possibility that the implantoplasty operator cannot guarantee the accuracy of the technique—it is established that the greater the number of samples, the higher the level of confidence.

The flexural strength tests were subjected to the following statistical analysis: Minitab version 17 (Minitab Inc., State College, PA, USA). The non-parametric Kruskal–Wallis test and Mann–Whitney U test were used to compare differences between conditions. Statistical significance was set at *p* < 0.05. In accordance with the ISO standard, the fatigue tests yield the number of cycles to failure at different applied cyclic loads; however, the standard does not require statistical analysis. In this study, a greater number of cycles were performed in order to ensure more accurate determination of the asymptote and to be able to determine the fatigue limit value using the MATLAB-Linear polyval-asymptotic program (Mathworks, Natick, MA, USA) (version 3.1).

## 3. Results

Table 1 shows the results of the elements analyzed in the simulation subjected to maximum stress and their corresponding deformation when force is applied under the conditions described in the methodology.

**Figure 6:**
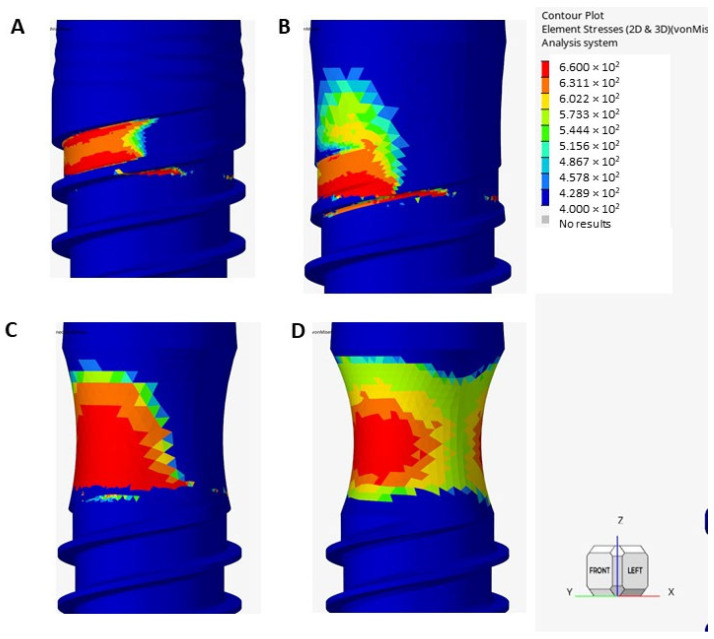
Distribution of von Mises stress of the four implants subjected to static loading. (**A**) Original implant. (**B**) Dental implant with implantoplasty of 0.2 mm. (**C**) Dental implant with implantoplasty of 0.4 mm. (**D**) Dental implant with implantoplasty of 0.6 mm.

Table 1 shows the results of the elements analyzed in the simulation subjected to maximum stress and their corresponding strain. As expected, the implant experiencing the highest von Mises stress is the one subjected to a 0.6 mm implantoplasty (663.7 MPa). This is due to the excessive reduction in cross-section, which causes a significant structural weakening of the implant. Interestingly, the implant with a 0.4 mm reduction presents a lower maximum stress (567.5 MPa) than the 0.2 mm group (571.2 MPa). This occurs because the 0.4 mm implantoplasty completely removes the upper thread—and along with it, the specific geometry that acts as a stress concentrator in the flexion area—distributing the load more evenly. Conversely, the 0.2 mm reduction leaves a partial thread structure that still acts as a critical stress raiser under bending loads. In Figure 6 the von Misses stresses are shown for the different dental implants subjected to a static force of 550 N. The maximum stress is located in the thread–body of the implant.

The stress-intensifying effect of the implant thread in the area of maximum stress, located between the flank and the bottom of the thread, can be observed. The maximum stress is distributed over a larger surface area in the treated implants, since the thread has been removed in that area.

Table 2 shows the flexural strength results for the different batches of implants studied, and Table 3 shows the *p*-values indicating statistically significant differences at *p* < 0.05. It can be observed that the flexural strength values for implants machined to 0.4 mm exhibit greater flexural strength in the static test than those machined to 0.2 mm. A significant decrease can also be observed when the implantoplasty reaches 0.6 mm, yielding values of 352 N, which are dangerous levels that human chewing forces may exceed. Regarding statistical significance, it can be seen that only the flexural strengths of the original implant and the 0.4 mm implant do not show statistically significant differences with a *p*-value < 0.05.

**Figure 7:**
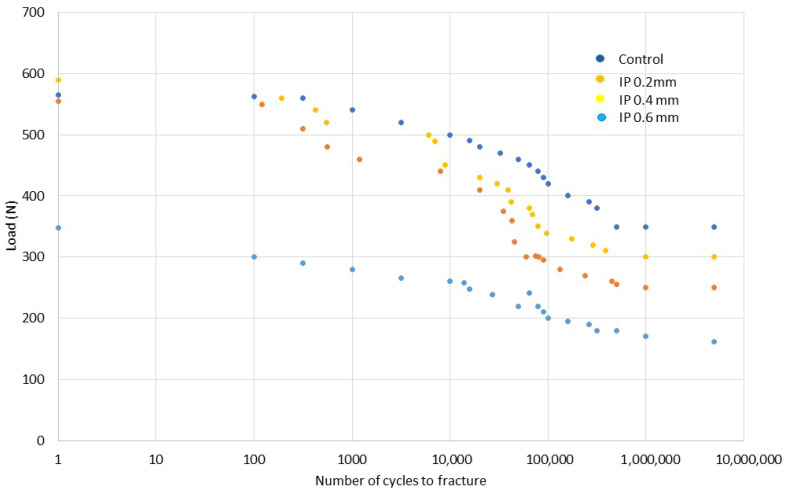
Load-number of cycles to fracture for different dental implants studied. Control, IP 0.2 mm: dental implant with implantoplasty of 0.2 mm. IP 0.4 mm: dental implant with implantoplasty of 0.4 mm. IP 0.6 mm dental implant with implantoplasty of 0.6 mm.

The fatigue results can be seen in Figure 7, which shows the load-number of cycles to fracture curve for the original dental implant, with implantoplasty reduction of 0.2, 0.4, and 0.6 mm of the section. Two clearly differentiated behaviors can be observed: those with low fatigue cycles up to 10,000 cycles approximately and those with high fatigue cycles which were tested until 10,000.000 cycles. It can be seen that the original implants have the highest fatigue life curve, followed by the dental implants subjected to 0.4 mm implantoplasty (i.e., leaving the implant without coils), followed by the dental implants subjected to 0.2 mm implantoplasty. For dental implants with a 0.6 mm reduction, the maximum bending load decreases by almost half and the fatigue curve is much lower than the other three.

Although the ISO standard [26] indicates that the fatigue limit is reached at 5 million cycles, in our case we have reached 10 million because a dental implant must be considered a device that must have mechanical reliability superior to the patient’s expected lifespan.

The fatigue limit results for each group of dental implants are shown in Table 4. The standard specifies that tests must be conducted for a minimum of 5 million cycles; however, in this study, we conducted tests up to 10 million cycles in order to establish the asymptote more clearly and determine the fatigue limit with greater accuracy. The asymptotic values obtained from the calculation yield the linear correlation coefficients shown in Table 5. As can be seen in all cases, the values are greater than 0.9, indicating good asymptotic behavior, which allows for the calculation of the fatigue limit shown in Table 5.

**Figure 8:**
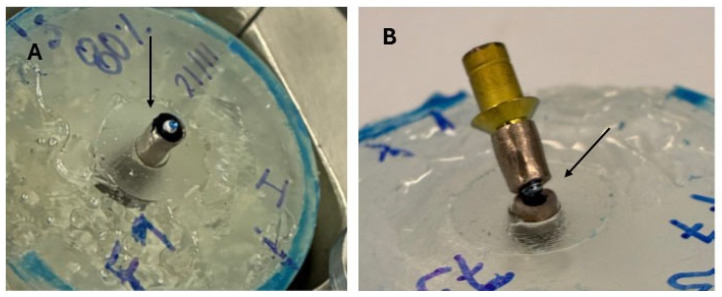
(**A**) Fracture for low number of cycles. The fracture is produced in the coronal region. (**B**) Fracture for high number of cycles. The fracture is produced in the internal connection zone. The arrows indicate the crack initiation zone.

**Figure 9:**
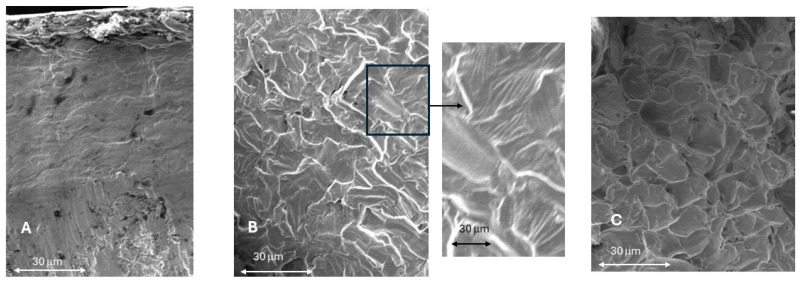
(**A**) Crack nucleation zone (×500). (**B**) Crack propagation (×500) and at higher magnification (×1500) where the stripes of the crack propagation can be observed. (**C**) Ductile fracture (×500).

Fractography studies show that in the first stage of fatigue up to 10,000 cycles, the fracture, corresponding to the highest loads, occurs in the coronal part, as can be seen in Figure 8A, and for a greater number of load cycles, the fracture occurs at the connection between the dental implant and the abutment, as can be seen in Figure 8B. This mode of fracture is appropriate for fatigue behavior as indicated by the ISO standard [26]. The fracture surfaces of the implants subjected to fatigue can be seen in Figure 9. Figure 9A shows the crack initiation zone, which has a polished surface due to the friction between the fracture faces. The nucleation zones correspond to machining defects on the surface caused by the machining of the implantoplasty. Figure 9B corresponds to the crack propagation zone, where the crack propagation marks can be seen at higher magnifications. Figure 9C corresponds to the final fracture of the implant, which occurred at the connection between the dental implant and the abutment. Figure 9B corresponds to the crack propagation zone where the crack propagation marks can be seen at higher magnifications. Figure 9C corresponds to the final fracture of the implant, which, as can be seen, has ductile behavior.

**Figure 10:**
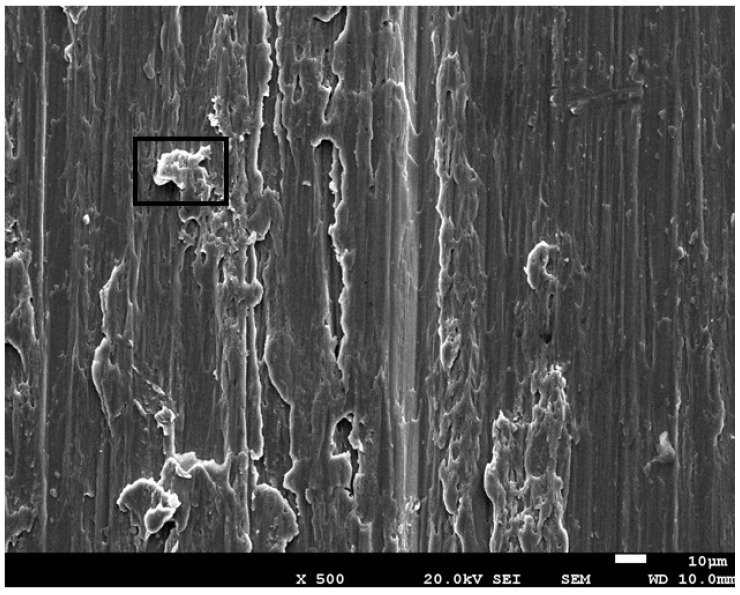

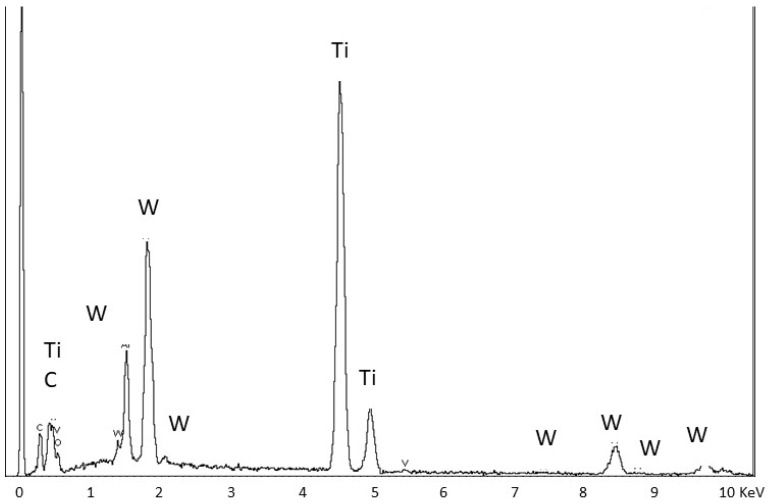
Surface of a titanium implant that has undergone implantoplasty (×500). The image shows residue from the drill used for machining. The X-ray energy dispersive microanalysis corresponds to the highlighted area along with the corresponding X-ray energy-dispersive microanalysis.

As mentioned, implantoplasty can cause damage to the surface of the dental implant, creating defects that may develop into small cracks, which can facilitate the initiation of fatigue cracks, as shown in Figure 10. The same figure also shows some residue from the tungsten carbide drill used, which can also alter the stress conditions on the surface.

**Figure 11:**
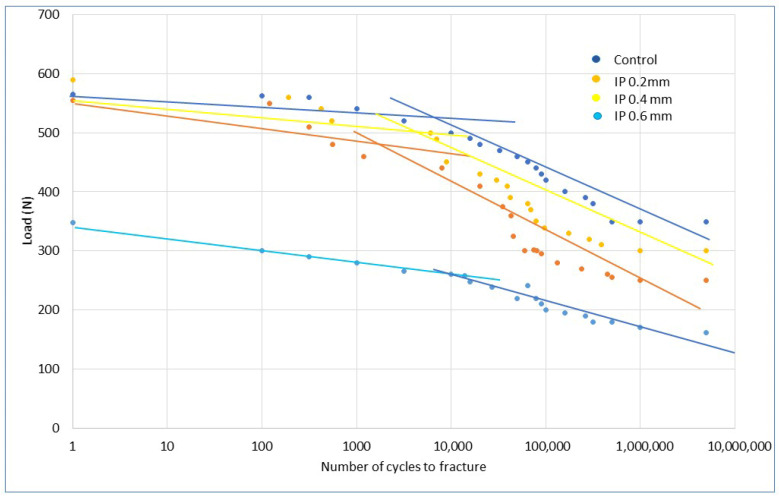
Load-number of cycles to fracture. The lines are the modelization of the fatigue behavior and the points are the experimental values obtained.

When performing the finite element simulation of fatigue behavior, straight lines with two different slopes are obtained for each of the implantoplasties performed. These correspond to low-cycle fatigue up to approximately 10,000 cycles and another straight line with a lower slope above this cycle value. It can be seen that the modeling performed is validated by the fatigue tests carried out. This comparison can be observed in Figure 11.

To validate the finite element results against the actual values obtained in the various tests, the experimental data points were fitted to the straight line corresponding to the computational factor, and the linear regression coefficient was calculated for the straight lines obtained from the mechanical behavior under low-cycle and high-cycle loading until fracture. Table 6 shows the coefficients, which in all cases are above 0.9, indicating a good correlation between the straight lines obtained from the simulation and the experimental data from the fatigue tests.

## 4. Discussion

Implantoplasty is an increasingly employed technique for the removal of biofilm, thereby aiming to halt the progression of bone loss associated with peri-implantitis. Previous studies have demonstrated that this procedure affects the corrosion resistance of the implant surface, as the coexistence of machined and non-machined areas may generate a galvanic couple that promotes corrosion when exposed to the physiological environment [32,33]. Furthermore, implantoplasty produces a smooth surface that is less favorable for fibroblast adhesion, and therefore may hinder the formation of soft tissue capable of contributing to the biological sealing of the implant. Similarly, osteoblast adhesion is not promoted, as it is well established that Sa roughness values between 0.9 and 1.6 µm are optimal for osteoblastic colonization [34]. Consequently, surfaces machined by implantoplasty present significant limitations for both soft and hard tissue regeneration [35,36]. However, roughness values below 0.088 µm disfavor bacterial colonization of titanium surfaces [37]. In addition, the release of titanium particles into the surrounding medium may trigger new inflammatory processes and immune responses. The cytotoxicity of titanium particles largely depends on their size and morphology, without considering the still poorly understood biological response to nanoparticles released into the patient’s oral cavity [17,18].

The present study aimed to evaluate the effect of controlled implant wall reduction, simulating implantoplasty, on the mechanical properties and in-service cyclic behavior of dental implants. The 30° bending tests revealed a reduction in fracture strength, with a marked decrease when wall reduction exceeded 0.4 mm, corresponding to the height of the implant thread. This finding can be explained by the significant reduction in the load-bearing wall thickness; as the resistant cross-section decreases, the maximum strength is correspondingly and sharply reduced. Fracture was consistently observed at the implant–abutment connection screw interface, which corresponds to the region with the minimum cross-sectional area [38].

One finding with significant clinical implications is that dental implants subjected to a 0.4 mm implantoplasty (corresponding to the width of the implant thread) have a longer fatigue life than those subjected to a 0.2 mm procedure. Specimens subjected to 0.4 mm implantoplasty—corresponding to complete thread removal—demonstrated the best fatigue performance, with a fatigue limit of approximately 350 N. In contrast, implants subjected to a 0.2 mm reduction exhibited inferior fatigue behavior, with a fatigue limit of approximately 275 N. These results, which may seem surprising, have been explained by the stress concentration caused by the screw thread on the body of the dental implant itself, which generates higher von Mises stresses for implants with 0.2 mm implantoplasty compared to those with 0.4 mm. These results, obtained from finite element simulation, have also been validated by fatigue testing. When implantoplasty eliminates the thread entirely (0.4 mm reduction), forming a cylindrical geometry, surface stress concentrations are minimized, resulting in improved fatigue behavior. In the fatigue life plot, it can be observed that the life curve for implants with 0.2 mm implantoplasty lies below the curve for 0.4 mm, indicating that experimentally we have found that all implants fracture earlier in the 0.2 mm implantoplasty group compared to the 0.4 mm group. Furthermore, in the fracture analyses of the implants, it can be seen that in all cases, the crack initiation zone in the implants with 0.2 mm implantoplasty is the connection between the screw and the body, at the junction of the dental implant and the abutment.

However, when machining extends beyond the thread into the implant body, the fatigue limit decreases to approximately 180 N. Under such conditions, fatigue fracture may occur between 10,000 and 1,000,000 cycles, and this fact should be taken into account to avoid using it in dental implants that are subjected to high mechanical loads. Clinicians should keep this in mind to prevent premature fractures.

Fractographic analysis showed that implants subjected to high loads and low-cycle numbers fractured at the coronal region. At lower applied loads, fracture occurred at the implant connection where the abutment screw is housed. This fracture mode is accepted by international standards for implant fatigue performance. Fracture occurring in the apical region is not acceptable [38,39]. This consideration is particularly relevant for clinicians managing bruxist patients or individuals with occlusal overload, in whom implantoplasty may adversely affect long-term implant performance [40].

Several studies have examined this same dental implant with both smooth and rough surfaces to determine the effect of surface roughness on the implant’s mechanical properties [41,42]. It has been found that the surface roughness achieved through sandblasting results in a higher fatigue limit than that of the smooth-surfaced implant. The fatigue limits are 350 N for the rough surface and 320 N for the smooth surface. The superior fatigue performance of the rough (original) implant compared to the smooth one, despite having the same dental implant design, is due to the residual compressive stress present in the rough implant. This surface compressive stress delays the onset of fatigue cracks and thus results in a longer fatigue life. In the case of the smooth dental implant, whose topography is similar to that of implants treated with implantoplasty, it has lower fatigue resistance due to the absence of residual compressive stress caused by the projection of abrasive particles onto the surface of the dental implant [43]. In the case of implantoplasty, the same would occur as with the smooth implant: the machining of the titanium causes the elimination of the compressive surface stress and, therefore, a reduction in fatigue life. These authors have previously studied compressive residual stresses in other publications [44,45] using the same dental implants and an identical implantoplasty protocol. It was determined using grazing-incidence X-ray diffraction with the Bragg–Bentano method that the residual stress decreased from −102 MPa to −15 MPa for original and implantoplasty-treated implants, respectively. It was determined that the compressive layer caused by alumina sandblasting is approximately 10 micrometers thick, meaning that 0.2-micrometer-thick compressive layer caused by the sandblasting has been removed by the implantoplasty. This fact must be taken into account in the analysis of fatigue life results.

It has also been determined that the microstructure of the titanium alpha phase does not vary between the original surfaces and those treated with implantoplasty. However, it is inevitable that the surface treated with surgical drills will develop surface defects, small cracks, and even inclusions of tungsten carbide burr debris, as can be observed in Figure 10, which can serve as sites conducive to crack initiation. Furthermore, it has been observed that these machined surfaces are susceptible to electrochemical corrosion, especially in areas where drill debris is present [32].

Another relevant factor in implantoplasty is the machining protocol. Initially, diamond or tungsten carbide burs are typically used to remove titanium and biofilm; subsequently, finer burs should be employed to minimize machining defects. Small surface fissures generated during the procedure may act as crack nucleation sites, as can inclusions, since they can also create stress concentration points. Therefore, it is essential not to exceed the dimensions of the implant body and to avoid introducing surface defects that could enhance stress concentration and fatigue crack propagation. A limitation of this study is that these defects were not taken into account in the modeling of the von Mises stress calculations.

Implantoplasty has relevant clinical implications in the management of peri-implantitis, particularly in non-regenerative surgical approaches [46,47]. Clinically, it has been associated with significant reductions in probing pocket depth (typically around 2–3 mm) and decreases in bleeding on probing, contributing to improved soft tissue health and disease stabilization over time. Survival rates of treated implants are generally high, often exceeding 85–90% at 3–5 years in observational studies [47]. Additionally, titanium particle release into peri-implant tissues has been documented, although its long-term biological impact remains uncertain. Implantoplasty may also lead to mucosal recession and esthetic concerns, particularly in anterior regions [48]. Overall, while it is an effective method for reducing bacterial load and improving clinical parameters, careful case selection is essential to balance its benefits against mechanical and biological risks.

To overcome these limitations—namely reduced mechanical reliability, particle release, and decreased corrosion resistance—alternative biofilm removal systems have been investigated. One approach involves electrolytic cleaning, using the implant as a cathode to generate hydrogen bubbles that detach biofilm from the surface. This electrolysis procedure is performed intraorally and is already applied in certain dental clinics. Experimental evidence indicates that this treatment does not impair mechanical properties while effectively cleaning the implant surface. This technique, known as GalvoSurge, is progressively being introduced into clinical practice [22]. Fatigue tests have been conducted on dental implants treated using the electrochemical technique, and no differences in fatigue limits were observed between treated and untreated implants. These results even show a slight increase in fatigue life, since, as Fonseca et al. indicate, the concentration of interstitial elements decreases, thereby improving the implants’ mechanical properties under cyclic loading conditions [49]. Clinical studies report significant improvements in key parameters, including a reduction in probing pocket depth from approximately 5.8 mm to around 3.1 mm, as well as marked decreases in bleeding on probing and suppuration [50]. In addition, radiographic and clinical evidence indicates substantial bone regeneration, with bone fill of about 2.7–2.8 mm and re-osseointegration occurring in up to 50% of treated implants [50,51]. These results suggest that the method not only controls infection but may also support true regenerative healing. Nevertheless, despite these encouraging findings, the available evidence remains limited in terms of sample size and long-term follow-up, and recurrence of peri-implantitis has been reported, highlighting the need for further well-designed clinical trials to confirm its long-term predictability [51].

Other approaches include high-pressure water jet systems and ozone projection systems aimed at oxidizing and eliminating biofilm-forming bacteria [52]. In principle, high-pressure water cleaning should not affect the cyclic mechanical properties of the dental implant, since the high-pressure water acts solely as a cleaning agent and does not remove material or introduce chemical elements that could affect its mechanical behavior. However, no mechanical studies have been conducted on this subject.

However, clinical evidence remains inconclusive, and further research is required to validate the effectiveness of bactericidal treatments that avoid implant machining. A novel device has been developed, the IMPACT implant planer, designed to machine contaminated implant surfaces intraorally and create a completely smooth surface, thereby promoting peri-implant tissue healing and potential re-osseointegration. This device allows precise control of the machining depth and may therefore help prevent excessive reduction beyond the implant body, particularly beyond the 0.4 mm threshold identified in the present study [53]. This treatment, which involves the loss of titanium from the implant, ensures reproducibility in implantoplasty procedures but will undoubtedly have a negative impact on the fatigue life of the dental implant. The authors of these studies have conducted clinical trials on the use of this implantoplasty technique, but there are no studies on the mechanical properties or the susceptibility to corrosion of the dental implant surface.

Finite element simulations were validated by the experimental results. The modeling accurately predicted stress concentration at the thread–implant body junction near the implant–abutment connection, explaining why a 0.4 mm reduction exhibited better mechanical behavior than a 0.2 mm reduction. Additionally, simulated fatigue curves showed good agreement with experimental fatigue data. Two fatigue regimes were identified: low-cycle fatigue and high-cycle fatigue, with a transition between approximately 1000 and 10,000 mechanical cycles.

This study has several limitations that should be considered. First, fatigue tests were conducted according to ISO 14801:2016 [26], which does not require testing under physiological conditions at 37 °C. It is well established that the physiological environment may reduce fatigue life by accelerating corrosion–fatigue processes in regions of high stored stress [54,55,56]. Therefore, the reported fatigue life values may vary under in vivo conditions. The surgical simulations (applied with a force of 10 N for 60 s) that we have obtained after numerous trials represent an attempt at standardization, but they do not fully reproduce the variability and potential damage associated with clinical implant surgery. For example, higher stresses can cause an increase in residual compressive stress on the surface, which will affect the material’s fatigue life, or lead to the formation of small surface cracks that may facilitate crack initiation. We have attempted to be as accurate as possible, but this is undoubtedly a limitation of the study.

Clinically, infected regions do not always involve the entire cylindrical surface exposed to the physiological environment, and surgeons may avoid machining areas where osseointegration is still present. Nevertheless, the present findings provide insight into the influence of implant body wall thickness reduction on fatigue behavior and underscore the importance of avoiding machining depths exceeding the thread height.

## 5. Conclusions

The experimental results showed a good agreement with the finite element simulations, supporting their potential to estimate stress distribution and fatigue behavior under the studied conditions. Overall, implantoplasty depth appears to play an important role in the mechanical reliability of the implant. The results suggest a relevant influence of thread removal, with 0.4 mm implantoplasty showing improved fatigue performance compared to 0.2 mm, possibly due to a reduction in stress concentration at the thread–implant junction. However, these findings should be interpreted with caution given the in vitro experimental conditions and the simplifications inherent to the numerical simulations. Within these limitations, the results suggest that implantoplasty depth should preferably not exceed the original thread height, as greater wall reduction may compromise cyclic mechanical behavior. This consideration may be particularly relevant in patients with increased occlusal loading, such as bruxers, although further validation under clinical conditions is required.

## Figures and Tables

**Table 1:** Flexural resistance and strain associated with the different models studied and the elements analyzed.

	Elements Analyzed	Von Misses Stress (MPa)	Strain
Original	518,759	566.2	0.02395
Implantoplasty 0.2 mm	444,365	571.2	0.02999
Implantoplasty 0.4 mm	373,916	567.5	0.02555
Implantoplasty 0.6 mm	353,345	663.7	0.13990

**Table 2:** Maximum flexural strength for each type of dental implant studied (n = 10).

	Maximum Flexural Strength
Original	591 ± 25
Implantoplasty 0.2 mm	555 ± 13
Implantoplasty 0.4 mm	582 ± 18
Implantoplasty 0.6 mm	352 ± 22

**Table 3:** Statistical significance values for each pair of dental implant batches.

	*p*
Original-IP 0.2 mm	0.0445 *
Original-IP 0.4 mm	0.0823
Original-IP 0.6 mm	0.0016 *
IP 0.2 mm-IP 0.4 mm	0.0486 *
IP 0.2 mm-IP 0.6 mm	0.0027 *
IP 0.4 mm-IP 0.6 mm	0.0012 *

The asterisks (*) indicate that there are statistically significant differences between them (*p* < 0.05).

**Table 4:** Fatigue limit results for each group of implants studied, obtained by determining the asymptote of the curve.

	Fatigue Limit (N)
Original	351
Implantoplasty 0.2 mm	255
Implantoplasty 0.4 mm	301
Implantoplasty 0.6 mm	185

**Table 5:** Linear regression coefficient for asymptotic behavior.

	r
Original	0.978
Implantoplasty 0.2 mm	0.996
Implantoplasty 0.4 mm	0.956
Implantoplasty 0.6 mm	0.917

**Table 6:** Linear regression coefficients of the lines obtained by simulation at low and high numbers of cycles for each condition.

	Low Number of Cycles	High Number of Cycles
Original	0.936	0.978
IP 0.2 mm	0.941	0.920
IP 0.4 mm	0.933	0.901
IP 0.6 mm	0.992	0.983

## Data Availability

The data that support the findings of this study are available from the corresponding author upon reasonable request.
